# Lack of Association between Interleukin-1*β* Gene Polymorphism (rs16944) and Chronic Periodontitis: From a Case-Control Studies to an Updated Meta-Analysis

**DOI:** 10.1155/2018/8287026

**Published:** 2018-12-11

**Authors:** Seoung-Jin Hong, Sang Wook Kang, Su Kang Kim, Young Sik Kim, Ju Yeon Ban

**Affiliations:** ^1^Department of Prosthodontics, Kyung Hee University Dental Hospital, Seoul, Republic of Korea; ^2^Department of Dental Pharmacology, College of Dentistry, Dankook University, Cheonan, Republic of Korea; ^3^Department of Biomedical Laboratory Science, Catholic Kwandong University, Gangneung, Republic of Korea; ^4^Management Research Institute, Kyung Hee University, Seoul, Republic of Korea

## Abstract

**Background:**

Interleukin-1*β* (IL-1*β*) plays an important role as a mediator of various inflammatory responses in chronic periodontitis. Several studies have investigated the potential relationship between IL-1*β* polymorphism (rs16944) and susceptibility to chronic periodontitis; inflammatory process is involved, but conclusions is still controversial.

**Objective:**

The aim of this study was to determine whether the IL-1*β* polymorphism (rs16944) is associated with susceptibility to chronic periodontitis.

**Material and Methods:**

For the case-control study, 51 patients with chronic periodontitis and 33 healthy control patients were recruited in the study. Genotyping was conducted by direct sequencing. SNPStats and SPSS 18.0 were used for the analysis of genetic data and to evaluate odds ratios, 95% confidence intervals, and *P* values; logistic regression models were used. And to perform meta-analysis, studies about IL-1*β* polymorphism (rs16944) and chronic periodontitis were searched in PubMed, Embase, Google Scholar, and Korean Studies Information Service System (KISS) electronic databases until July 2017.

**Results:**

In our case-control study, no significant relationship was revealed between IL-1*β* polymorphism (rs16944) and chronic periodontitis (*P* > 0.05 in each model). When combined with the previous studies in the meta-analysis, the result was not associated with chronic periodontitis in any of the models (CC vs. CT + TT: OR = 0.97, 95% CI = 0.762–1.246; CC + CT vs. TT: OR = 0.90, 95% CI = 0.658–1.232; and C vs. T: OR = 0.93, 95% CI = 0.774–1.128). The subgroup analysis stratified by ethnicity showed a weak association between the IL-1*β* polymorphism (rs16944) and chronic periodontitis in the Caucasian population (recessive model, OR = 1.34, 95% CI = 1.017–1.758, *P* = 0.037).

**Conclusion:**

Evidences from a case-control study and the meta-analysis suggest that IL-1*β* polymorphism (rs16944) is not associated with susceptibility to chronic periodontitis.

## 1. Introduction

Periodontitis, one of the most common infectious diseases, is the major cause of tooth extraction in the elderly population [1]. According to the Organisation for Economic Co-operation and Development (OECD), in 2014, the elderly accounted for 25.06% of the population in Japan, 21.45% in Germany, 21.25% in Italy, 19.94% in Finland, 18.31% in Spain, and 12.66% in Korea and this number is continuously increasing [2]. With the increase of elderly population, the prevalence and social burden of periodontitis increase and demands on the efficient treatment of the disease also increase [3]. Thus, the importance of risk factor-based preventive strategies of periodontitis has been getting attention. Periodontitis is an inflammatory disease caused by several factors, including both genetic and environmental risk factors. Many researchers agree that the susceptibility to periodontal disease is at least partially influenced by a genetic factor [4].

The interleukin-1 beta (IL-1*β*) gene located at 2q14 encodes the IL-1*β* protein which plays an important role as a mediator in a variety of inflammatory response as well as in bone metabolism [5, 6]. A previous study showed that the amount of IL-1*β* mRNA expression is much higher in a group affected by chronic periodontitis than in the control group [7]. At the IL-1*β* transcription site, three polymorphisms at base pair positions +3954/3953 (C → T, rs1143634), −511 (C → T, rs16944), and −31 (T → C, rs1143627) caused transitions between C and T [8–10]. IL-1*β* polymorphism (rs16944) is one of the most commonly investigated polymorphism regarding susceptibility to chronic periodontitis. Kornman et al. reported on the relationship between the IL-1*β* polymorphism (rs16944) and periodontal diseases in a population of European descent for the first time in 1997 [11]. Since then, many studies investigated the relationship between IL-1*β* polymorphism (rs16944) and chronic periodontitis, with many results that do not coincide and remain debatable. The discrepancy in results may be due to heterogeneity between studies, as well as small sample sizes. To overcome issues due to small sample sizes and error from inadequate statistical power, meta-analysis is performed. To obtain more reliable results on the relationship between the IL-1*β* polymorphism (rs16944) and the susceptibility to chronic periodontitis, we performed a case-control study and meta-analysis with all eligible studies.

The purpose of this study was to evaluate the association of IL-1*β* polymorphism (rs16944) and the susceptibility to chronic periodontitis from a new case-control study and then to further perform an updated meta-analysis to derive a more precise conclusion.

## 2. Materials and Methods

### 2.1. Participants for Case and Control in the Present Study

To investigate the relationship between IL-1*β* gene polymorphism (rs16944) and the susceptibility to chronic periodontitis, 51 patients with chronic periodontitis and 33 nonperiodontitis controls were recruited. The criteria for diagnosis for chronic periodontitis and control groups are described in Kang et al. [12]. This study was approved by the Institutional Review Board of College of Dentistry, Dankook University, Cheonan, Republic of Korea (IRB number: H-1204/004/001). Written consent forms were received from all subjects.

### 2.2. DNA Extraction and Genotyping

A previous study reported that oral epithelial cells near teeth in patients with gingivitis and periodontitis underwent changes such as proliferation, DNA damage, or apoptosis [13]. Collection of genomic DNA by buccal swabs is a simple and low-cost means and showed an adequate polymerase chain reaction results for genetic studies [14]. Thus, buccal swabs were collected to obtain genomic DNA from the patients [15]. Briefly, the patients were asked to wash their mouth with water, and we confirmed that no substance obstructed the DNA typing methods before collecting the cells from the inside of a patient's mouth, physically. For the buccal region of each cheek, two sterilized swabs were used to collect cells. A swab scraping was conducted over 10 times for collecting sufficient amount of epithelial cells from the side of the cheek. Each DNA sample was extracted according to the protocol of the DNA isolation reagent kit as previously described (Roche, Mannheim, Germany) [16].

### 2.3. Statistical Analysis for Case and Control in the Present Study

The Hardy–Weinberg equilibrium (HWE) was estimated using SNPStats (http://bioinfo.iconcologia.net/SNPstats) in the control group. Genotype models (codominant, dominant, recessive, and log-additive models) were applied to determine relationship between differences in genotype frequencies between the chronic periodontitis group and the control group. Logistic regression was used for the analysis. SNPStats and SPSS 18.0 (SPSS Inc., Chicago, IL, USA) were used for the statistical analysis. Logistic regression was conducted to evaluate the odds ratio (OR), 95% confidence intervals (CI), and *P* value. For statistical tests, the level of significant *P* value was set at 0.05.

### 2.4. Meta-Analysis

To further investigate the association of IL-1*β* polymorphism (rs16944) with chronic periodontitis, a meta-analysis combining published literature and our current study was performed. Studies about IL-1*β* polymorphism (rs16944) and chronic periodontitis were searched in electronic databases including PubMed, Embase, Google of Scholar, and Korean Studies Information Service System (KISS) up to July 2017 using the keywords “Interleukin-1*β*”, “IL1B”, “rs16944”, or “IL1B-511”, AND “polymorphism”, “polymorphisms”, or “variant” AND “alveolar bone loss”, “periodontitis”, or “periodontal disease”. First, we screened the titles and abstracts, and then examined the full text of screened articles. Some studies were included by manual search from references in related original studies or review articles.

Inclusion criteria were the following: (1) the study about the association between IL-1*β* polymorphism (rs16944) and chronic periodontitis, (2) a case-control study, and (3) sufficient genotype and allele distribution data of the IL-1*β* polymorphism (rs16944) in the control group and the chronic periodontitis group to calculate an odds ratio (OR) with a 95% confidence interval (CI). The data of the first author's name, published year, country, ethnicity, sample size of the control and chronic periodontitis, and genotype frequencies of IL-1*β* polymorphism (rs16944) in the control and chronic periodontitis groups were extracted from the included studies.

Statistical analysis was performed by meta-analysis software (Comprehensive Meta-Analysis; Biostat Inc., NJ, USA). The random effects model or the fixed effects model was selected according to the degree of heterogeneity. The *I*^2^ statistic for inconsistency and the *χ*^2^ distribution of Cochran *Q* statistic were used to assess heterogeneity across studies. A *χ*^2^ test-based *Q* statistic test and a *I*^2^ test were performed to assess heterogeneity of the study. The *P* value less than 0.05 of *Q* was considered to be statistically significant. The fixed effects model was adopted if the result of the *Q* test was *P* > 0.05 or *I*^2^ statistic was <50%, which indicated the statistically no significant heterogeneity in the meta-analysis. The random effects model was used when significant heterogeneity was detected among the included studies. Publication bias was evaluated by a funnel plot and Egger's test.

## 3. Results and Discussion

### 3.1. Case-Control Study

In the present study, association of the single nucleotide polymorphism of the IL-1*β* gene with susceptibility to chronic periodontitis was investigated.  Table 1 shows analysis of genotype of IL-1*β* polymorphism (rs16944) with chronic periodontitis. In the control group, genotype distributions for IL-1*β* polymorphism (rs16944) were in HWE (*P* = 0.178). Frequencies of C/C, C/T, and T/T genotypes were 37.5%, 37.5%, and 25% in the control group, and 22%, 64%, and 14% in the chronic periodontitis group, respectively (Table 1).

Any association between IL-1*β* polymorphism (rs16944) and chronic periodontitis was not observed. In the genotype analysis of the IL-1*β* polymorphism, all models (codominant, dominant, recessive, and log-additive models) showed no significant association with chronic periodontitis (*P* > 0.05).

### 3.2. Study Selection for Meta-Analysis

**Figure 1:**
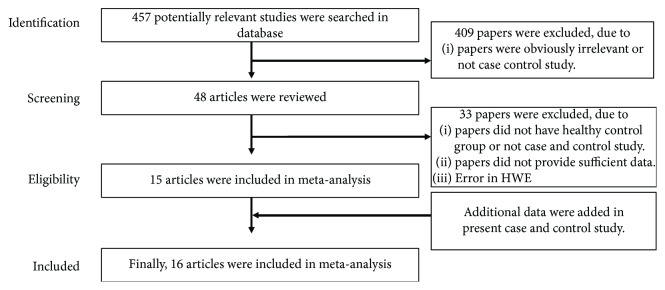
Flow chart illustrating the search strategy used for this meta-analysis to identify studies that evaluate the relationship between IL-1*β* polymorphism (rs16944) and susceptibility to chronic periodontitis.

A total of 457 studies were screened using the mentioned search strategy. After deleting duplicates, a record screen was performed and 16 case-control studies were finally selected [17–31]. The search process and strategy of study selection are showed in Figure 1. We reviewed 48 articles, and 30 articles were omitted because they did not have a healthy control group or not a case-control study or did not provide sufficient data. Among them, 3 studies were excluded because of inconsistency with HWE.

### 3.3. Characteristics of Eligible Studies

The characteristics of selected studies in meta-analysis, including our case-control study, are showed in Table 2 [31]. A total of 16 studies were included in the present meta-analysis. All studies were case-control studies. The 16 articles included 1540 patients with chronic periodontitis and 2472 healthy control subjects. Of the 16 included studies, 9 studies were conducted in Asian population and 7 studies were conducted in Caucasian population. All the studies conformed to HWE (*P* > 0.05).

### 3.4. Quantitative Synthesis

**Figure 2:**
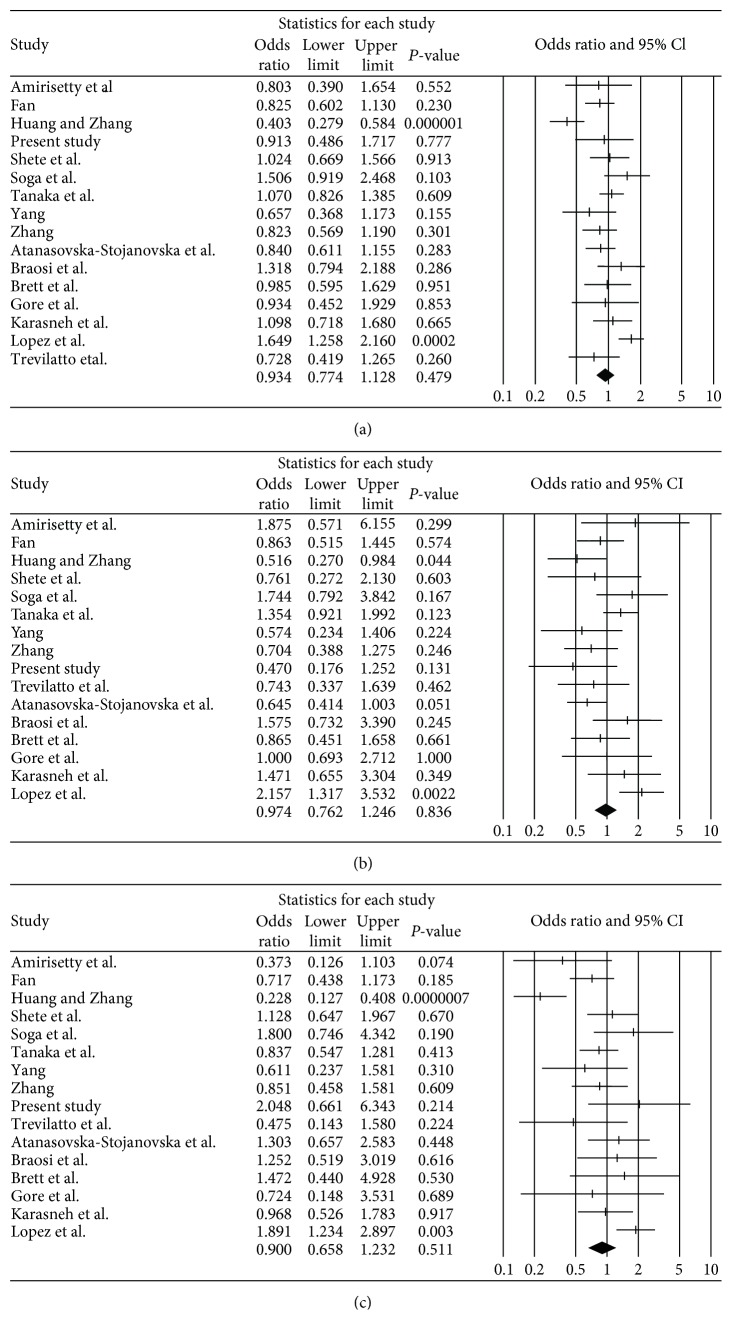
Odds ratio and 95% CI of individual and pooled data for the IL-1*β* polymorphism (rs16944) and susceptibility to chronic periodontitis. (a) C allele vs. T allele. (b) C/C genotype vs. C/T genotype + T/T genotype. (c) C/C genotype + C/T genotype vs. T/T genotype.

We evaluated the association between susceptibility to chronic periodontitis and IL-1*β* polymorphism (rs16944) (Table 3). There was no association observed between chronic periodontitis and IL-1*β* polymorphism (rs16944) in the allele, dominant, and recessive models (OR = 0.93, 95% CI = 0.774–1.128, *P* = 0.48 in the allele model; OR = 0.97, 95% CI = 0.762–1.246, *P* = 0.84 in the dominant model; and OR = 0.90, 95% CI = 0.658–1.232, *P* = 0.51 in the recessive model, Table 3 and Figure 2).

**Figure 3:**
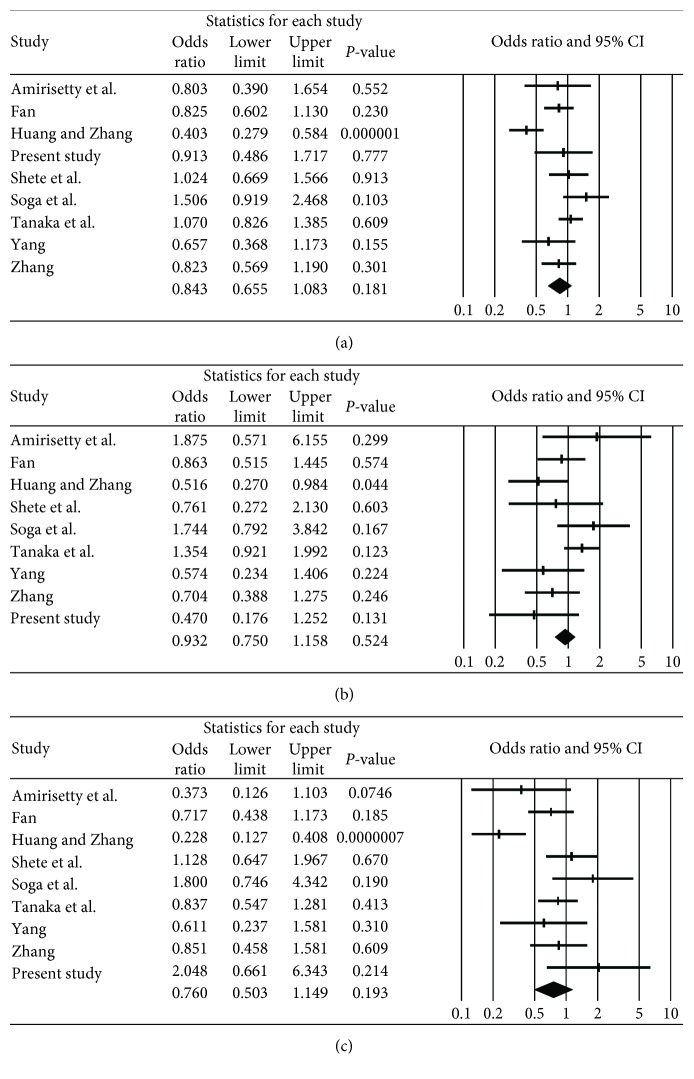
Odds ratio and 95% CI of individual and pooled data for the IL-1*β* polymorphism (rs16944) and susceptibility to chronic periodontitis in Asian population. (a) C allele vs. T allele. (b) C/C genotype vs. C/T genotype + T/T genotype. (c) C/C genotype + C/T genotype vs. T/T genotype.

In the subgroup analysis of the Asian population, there was also no association observed between chronic periodontitis and IL-1*β* polymorphism (rs16944) in the allele, dominant, and recessive models (OR = 0.84, 95% CI = 0.655–1.083, *P* = 0.18 in the allele model; OR = 0.93, 95% CI = 0.750–1.158, *P* = 0.52 in the dominant model; and OR = 0.76, 95% CI = 0.503–1.149, *P* = 0.19 in the recessive model, Table 3 and Figure 3).

**Figure 4:**
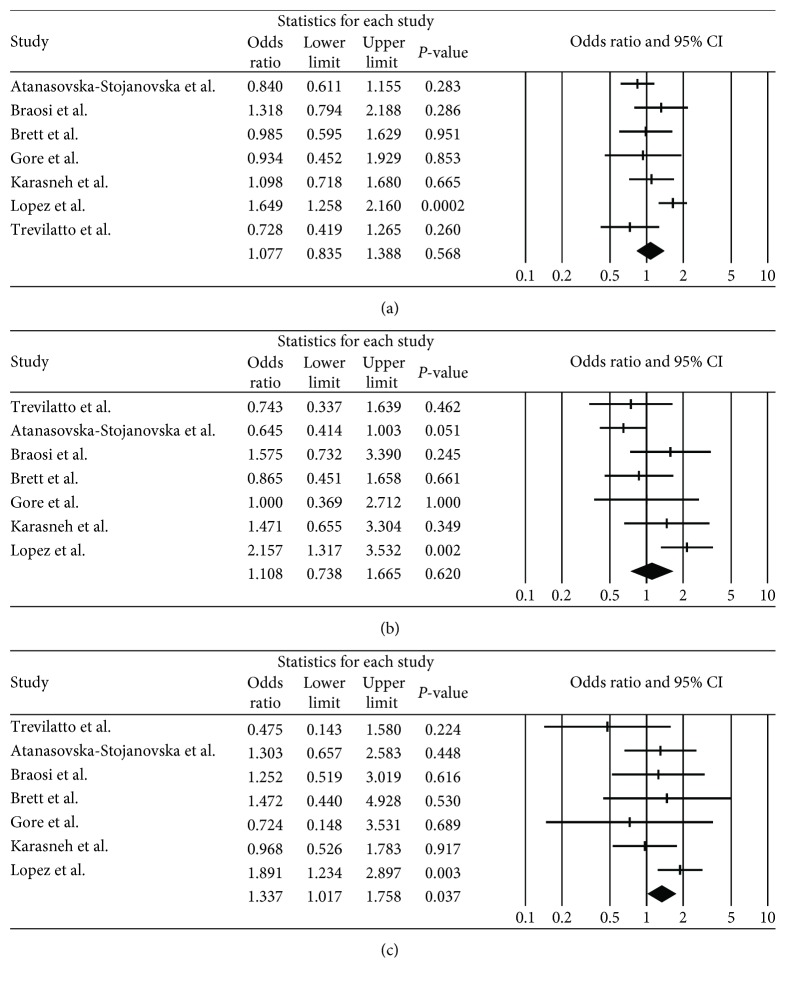
Odds ratio and 95% CI of individual and pooled data for the IL-1*β* polymorphism (rs16944) and susceptibility to chronic periodontitis in Caucasian population. (a) C allele vs. T allele. (b) C/C genotype vs. C/T genotype + T/T genotype. (c) C/C genotype + C/T genotype vs. T/T genotype.

IL-1*β* polymorphism (rs16944) showed a weak association with chronic periodontitis in the Caucasian population (OR = 1.34, 95% CI = 1.017–1.758, *P* = 0.037 in the recessive model). However, association between IL-1*β* polymorphism (rs16944) and chronic periodontitis was not observed in the allele (OR = 1.08, 95% CI = 0.835–1.388, *P* = 0.27) and dominant (OR = 1.11, 95% CI = 0.738–1.665, *P* = 0.83) models (Table 3 and Figure 4). Consequently, it is difficult to define significant association between IL-1*β* polymorphism (rs16944) and chronic periodontitis.

**Figure 5:**
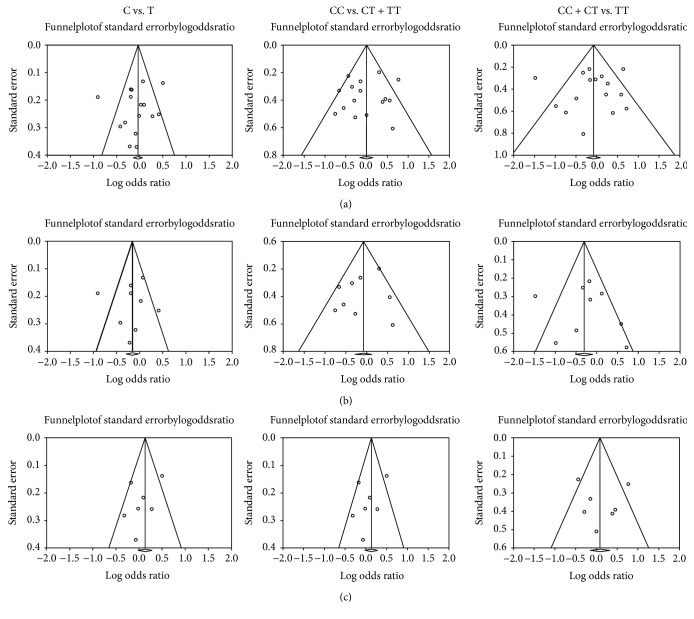
Funnel plot of publication bias for the IL-1*β* polymorphism (rs16944) and susceptibility to chronic periodontitis. (a) Funnel plot in all population. (b) Funnel plot in Asian population. (c) Funnel plot in Caucasian population.

Table 3 and Figure 5 show the analysis of funnel plots for publication bias. Funnel plots were graphed by standard error plotted against the OR for each study. No evidence of asymmetry was indicated in the funnel plots (*P* > 0.05), and Egger's linear regression test showed that there was no publication bias (*P* > 0.05).

### 3.5. Discussion

It is not too much to emphasize the importance of existence of teeth in one's life. The presence of teeth is closely connected with one's daily activities and quality of life (QoL) [32]. Tooth loss is a critical factor in the impairment of QoL, and location and distribution of missing tooth plays a major role in the severity of the impairment [33]. Therefore, the preservation of natural teeth is very important. It contributes to a positive body image and increase in self-worth and positively influences QoL [34]. Likewise, proper dental prostheses or implants could compensate for tooth loss to improve QoL [35]. To this end, both social and individual healthcare budgets in the world are increasing continuously. According to the Korean National Health Insurance Service (NHIS), periodontitis is one of the most common oral diseases in Korea and the cost of health insurance exhibits a growing trend with NHIS expenditure [36]. Thus, the need for efficient prediction and risk factor-based periodontitis preventive strategies is rapidly growing both in clinical and economical terms.

IL-1*β* is expressed most abundantly in blood mononuclear cells and mediates host response [37]. IL-1*β* plays an important role in inflammation, regulating inflammatory pain hypersensitivity by modulating COX2 expression [38]. CD4+ T helper cells, important for host defense and immunity, selectively express Th17, even in the absence of TGF-beta signaling. IL-1*β* can also participate in this process [39]. Because of the role of IL-1*β* in these inflammations, there have been many studies on inflammatory diseases and polymorphisms of the IL-1*β* gene. IL-1*β* polymorphism may be associated with an increased risk of hypochlorhydria and gastric cancer by altering the level of IL-1*β* expression due to *Helicobacter pylori* infection [40]. Studies on inflammatory bowel disease in South Africa found more IL-1*β* gene mutations in the patient group than in the control group [41]. The IL-1*β* polymorphism (rs16944), one of the single nucleotide polymorphisms of IL-1*β*, has been reported to be associated with various diseases, such as diabetic nephropathy in Korean type 2 diabetic patients [42], Parkinson's disease in Finnish patients [43], and Behçet's disease in Turkish patients [44].

Therefore, there have been many studies on periodontitis as one of the inflammatory diseases. Ever since the first reported study on the association between the IL-1*β* polymorphism (rs16944) and chronic periodontitis in a population of European descent [11], many studies have been undertaken to explore this association. However, results of individual studies have been inconclusive. There was a study suggested IL-1*β* polymorphism (rs16944) as a risk indicator for chronic periodontitis in Brazilians [28]. In contrast, another study in Japanese women showed an association between the genotype of IL-1*β* polymorphism (rs16944) and reduced risk of periodontitis [27]. A recent meta-analysis study in the Chinese population suggested association between IL-1*β* polymorphism (rs16944) variants and the risk of periodontitis [45].

In the present study, we have collected the previous studies conducted on the association between IL-1*β* polymorphism (rs16944) and chronic periodontitis. Our study included 1540 patients with chronic periodontitis and 2472 healthy control subjects in 16 articles. The result shows that the development of chronic periodontitis is not associated with IL-1*β* polymorphism (rs16944), except in the case of Caucasian population, where a weak statistical significance in the recessive model was detected (*P* = 0.037). Our present result for host response might partially explain this difference.

The purpose of our study was to find a significant relationship between IL-1*β* polymorphism (rs16944) and the chronic periodontitis, but our results failed to show the association. These findings should be interpreted with caution and have some limitations. We could not investigate various ethnic distributions because all studies included only Caucasian and Asian populations. Though environmental or habitual elements are key factors in the risk of chronic periodontitis and the pathophysiological processes are very complex, we cannot but consider only one genetic factor in this meta-analysis. With this regard, we have added a new original case-control study to the meta-analysis and have found some more accurate results by removing some papers outside of the HWE. The ethnic-specific genotypes CC, CT, and TT of rs16944 are known as 0.416, 0.451, and 0.133 in Caucasian, 0.279, 0.535, and 0.186 in Chinese, and 0.290, 0.465, 0.244 in the Japanese population, respectively. The genotype in our case-control study in the Korean population was 0.375, 0.375, and 0.250. These differences among genotypes are likely to be the reason for the different results between ethnicities.

## 4. Conclusion

We evaluated the association between IL-1*β* polymorphism (rs16944) and chronic periodontitis in the present case and control study. We observed that IL-1*β* polymorphism (rs16944) was not associated with chronic periodontitis in the Korean population. We also evaluated the relationship between IL-1*β* polymorphism (rs16944) and chronic periodontitis using meta-analysis. A statistically meaningful association was detected with IL-1*β* polymorphism (rs16944) in the Caucasian population, but it is difficult to describe a strong association with chronic periodontitis due to insufficient statistical power. If more results in various populations would be accumulated in further studies, the relation between IL-1*β* polymorphism (rs16944) and chronic periodontitis would be clarified and that would help improving clinical prognosis.

## Figures and Tables

**Table 1:** Analysis of genotypes of IL-1*β* polymorphism (rs16944) with chronic periodontitis.

Model	Genotype	Control *N* (%)	Chronic periodontitis *N* (%)	OR (95% CI)	*P*
Codominant	C/C	12 (37.5%)	11 (22.0%)	1	0.06
	C/T	12 (37.5%)	32 (64.0%)	2.91 (1.01–8.34)	
	T/T	8 (25.0%)	7 (14.0%)	0.95 (0.26–3.51)	

Dominant	C/C	12 (37.5%)	11 (22.0%)	1	0.13
	C/T-T/T	20 (62.5%)	39 (78.0%)	2.13 (0.80–5.67)	

Recessive	C/C-C/T	24 (75.0%)	43 (86.0%)	1	0.21
	T/T	8 (25.0%)	7 (14.0%)	0.49 (0.16–1.51)	

Log-additive	—	—	—	1.10 (0.57–2.14)	0.77

OR: odds ratio; CI: confidence interval.

**Table 2:** Information of eligible studies included in the meta-analysis.

First author	Disease	Year	Country	Ethnicity	Chronic periodontitis			Control			HWE in the control
					CC	CT	TT	CC	CT	TT	
Amirisetty et al.	Chronic periodontitis	2014	India	Asian	9	6	14	6	17	8	0.57
Atanasovska-Stojanovska et al.		2013	Macedonia	Caucasian	42	60	12	143	118	40	0.05
Braosi et al.		2012	Brazil	Caucasian	24	28	12	16	29	13	0.98
Brett et al.		2005	United Kingdom	Caucasian	27	26	4	51	39	10	0.53
Fan		2009	China	Asian	41	78	58	36	67	36	0.67
Gore et al.		1998	United States	Caucasian	13	15	4	13	16	3	0.54
Huang and Zhang		2004	China	Asian	25	58	99	21	49	19	0.33
Karasneh et al.		2011	Jordan	Caucasian	19	44	37	11	40	29	0.63
Lopez et al.		2009	Chile	Caucasian	58	117	49	29	107	72	0.28
Shete et al.		2010	India	Asian	7	48	45	9	43	48	0.89
Soga et al.		2003	Japan	Asian	21	33	10	14	34	16	0.61
Tanaka et al.		2014	Japan	Asian	45	54	32	284	518	217	0.50
Trevilatto et al.		2011	Brazil	Brazilian	22	35	12	17	23	4	0.33
Yang		2008	China	Asian	11	20	13	18	21	10	0.40
Zhang		2009	China	Asian	24	54	25	38	61	27	0.78
Present study		2017	Korean	Asian	11	32	7	12	12	8	0.18

HWE: Hardy–Weinberg equilibrium.

**Table 3:** Overall analysis of the association between IL-1*β* polymorphism (rs16944) and susceptibility to chronic periodontitis.

Models	Group	Heterogeneity		Model	OR	95% CI	*P*	Egger's *P*
		*P*	*I* ^2^					
C vs. T	All	<0.001	63.39	Random	0.93	0.774–1.128	0.48	0.45
	Asian	0.001	68.53	Random	0.84	0.655–1.083	0.18	0.85
	Caucasian	0.028	57.71	Random	1.08	0.835–1.388	0.57	0.27

CC vs. CT + TT	All	0.008	51.83	Random	0.97	0.762–1.246	0.84	0.62
	Asian	0.066	45.47	Fixed	0.93	0.750–1.158	0.52	0.42
	Caucasian	0.016	61.52	Random	1.11	0.738–1.665	0.62	0.83

CC + CT vs. TT	All	<0.001	67.01	Random	0.90	0.658–1.232	0.51	0.76
	Asian	0.001	70.45	Random	0.76	0.503–1.149	0.19	0.77
	Caucasian	0.313	15.32	Fixed	1.34	1.017–1.758	**0.037**	0.83

OR: odds ratio; CI: confidence interval. Bold numbers indicate a significant *P* value.

## Data Availability

Genotyping results of subjects participating in the case-control study are shown in Table 1. In addition, additional data used to support the findings of this study are available from the corresponding author upon request. The data supporting this meta-analysis are from previously reported studies and datasets, which have been cited within the text as references [15–29].
